# Case Report: Anomalous drainage vein sampling for diagnosing aldosterone-producing lesions undetectable by segmental adrenal venous sampling in a two-case series

**DOI:** 10.3389/fradi.2025.1567779

**Published:** 2025-06-10

**Authors:** Hiromitsu Tannai, Sota Oguro, Hiroki Kamada, Yuta Tezuka, Yoshikiyo Ono, Kei Omata, Kei Takase

**Affiliations:** ^1^ Department of Diagnostic Radiology, Tohoku University Hospital, Sendai, Japan; ^2^ Department of Diabetes, Metabolism and Endocrinology, Tohoku University Hospital, Sendai, Japan

**Keywords:** adrenal venous sampling, aldosterone-producing adenoma, primary aldosteronism, hypertension, computed tomography

## Abstract

Adrenal vein sampling (AVS) is the gold standard for subtyping primary aldosteronism (PA). However, through conventional AVS, unilateral PA may be misdiagnosed as bilateral PA. Compared with conventional AVS, segmental AVS with additional sampling in adrenal tributaries can detect aldosterone-producing adenomas (APAs) with higher sensitivity. Herein, we describe two cases wherein high aldosterone levels were not detected through initial segmental AVS but were identified in anomalous drainage veins during the second AVS session. In Case 1, computed tomography (CT) during left adrenal arteriovenography revealed a fine renal capsular vein connecting an adrenal nodule to the third lumbar vein. Sampling in this vein during the second AVS revealed high aldosterone levels. The surgical specimen showed the presence of an 11 mm APA. Furthermore, Case 2 presented with bilateral small adrenal nodules; bilateral renal capsular vein sampling was performed during the second AVS session. The samples from the renal capsular vein connected to the renal vein revealed considerably high aldosterone levels. Left adrenalectomy revealed the presence of a 6 mm aldosterone-producing nodule. These cases highlight the importance of anomalous drainage vein sampling, the limitation of conventional and segmental AVS in diagnosing PA, and the utility of CT during adrenal arteriovenography for estimating the drainage route.

## 1 Introduction

Adrenal vein sampling (AVS) is the gold standard for subtyping primary aldosteronism (PA), a common cause of secondary hypertension (1, 2). Most subtypes include unilateral PA due to aldosterone-producing adenoma (APA) and bilateral PA due to idiopathic hyperaldosteronism or bilateral hyperaldosteronism. Some cases of unilateral APA could be misdiagnosed as bilateral PA through conventional AVS (cAVS) in which samples are obtained from both adrenal veins (3–7).

Compared with cAVS, segmental AVS (sAVS) in which samples are obtained from adrenal tributaries using a microcatheter can detect APA and small aldosterone-producing nodules (APNs) with higher sensitivity (6). However, in rare cases, aldosterone levels may only be detected in anomalous drainage veins (ADV). The sampling techniques in the ADV are not well-known, including the identification of target blood vessels, the choice of catheters, the specific sampling positions, and the expected results. We successfully detected elevated aldosterone levels through this route. Herein, we reported the details of these challenging cases. In Case 1, a 55-year-old woman was diagnosed with hypertension (141/107 mmHg) and hypokalemia (serum potassium level, 3.2 mmol/L) during a health checkup and subsequent further examination at a local hospital. She had a medical history of depression, asthma, and sleep apnea syndrome. Laboratory tests revealed elevated plasma aldosterone concentration (PAC; 17.9 ng/dl) and low plasma renin activity (PRA; 0.5 ng/ml/h), resulting in a high aldosterone-to-renin ratio (ARR; 35.8) with daily administration of amlodipine (5 mg) at a prior hospital. Captopril challenge test showed an ARR of 164 after 90 min, and saline infusion test revealed a PAC of 28.7 ng/dl after 240 min; thus, confirming PA (2). In Case 2, a 57-year-old man with a 7-year history of hypertension presented with hypokalemia (serum potassium level, 2.9 mmol/L) at a local doctor. PA screening test is positive. At the time of admission, the PAC was 10.6 ng/dl, PRA was ≤0.2 ng/ml/h, ARR was ≥53 with daily oral administration of amlodipine (10 mg) and potassium chloride (45 mmol) (1, 2). Captopril challenge test showed an ARR of 45.2 after 90 min, and saline infusion test revealed a PAC of 6.0 ng/dl after 240 min; thus, confirming PA (2).

## 2 Case description

### 2.1 Case 1

**Figure 1:**
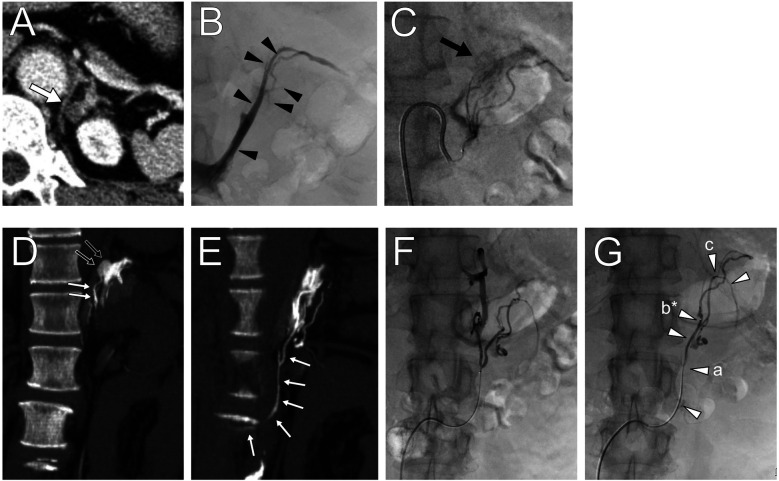
Contrast-enhanced computed tomography (CT) images showing **(A)** a low-density left adrenal nodule (white arrow). **(B)** Left adrenal venography image highlighting the locations of segmental adrenal venous sampling in the tributaries (black arrowheads). **(C)** The left inferior adrenal arteriography depicting a tumor stain. **(D,E)** CT during adrenal arteriovenography revealed tumor enhancement (black small arrows) and a drainage vein (white small arrows) connected to the third lumbar vein. **(F,G)** Anomalous drainage vein sampling was performed at several sites (white arrowheads) from the vein. The letters a–c in parenthesis shown in Table 1 correspond to sampling points, and those marked with an asterisk indicate the maximum aldosterone concentration.

Before AVS, precontrast and dynamic contrast-enhanced computed tomography (CT) were performed, and an 11 mm adrenocortical adenoma was detected in the left adrenal gland (Figure 1A). In segmental AVS (sAVS), samples were obtained from both adrenal central veins and tributaries to localize excess aldosterone, as previously described (6). Adrenocorticotropic hormone (ACTH) was administered at an initial bolus dose of 200 µg, followed by a continuous intravenous infusion at a rate of 50 µg/h starting 30 min later (6, 7).

**Table 1:** Adrenal venous sampling results in Cases 1 and 2.

Sampling points	First AVS results			Second AVS results		
	PAC, ng/dl	PCC, µg/dl	A/C	PAC, ng/dl	PCC, µg/dl	A/C
Case 1						
LAV_CV_	472	422	1.1	2,742	251	**10.9**
LAV_CMT_	264	198	1.3	1,068	106	**10.1**
RAV	152	322	0.47	–	–	–
EIV	42.7	17.8	2.4	32.2	12.9	2.5
			LI_CV_ 2.4, LI_CMT_ 2.8			LI_CV_ 23.2, LI_CMT_ 21.5
LTVs	154–690	240–433	0.5–2.2	–	­–	–
Lt RenCV, proximal (a)	–	–	–	8,921	87.2	**102.3**
Lt RenCV, middle (b)	–	–	–	34,125	223	**146**
Lt RenCV, distal (c)	–	–	–	41.9	16	**2.62**
RTVs	101–257	225–561	0.24–0.46	–	–	–
Case 2						
LAV_CV_	147	574	0.26	97.1	328	0.3
LAV_CMT_	129	181	0.71	117	107	1.09
RAV	150	659	0.23	139	733	0.19
EIV	28.6	18.6	1.54	28.6	18.6	1.54
			LI_CV_ 1.1, LI_CMT_ 3.1			LI_CV_ 1.8, LI_CMT_ 5.7
LTVs	274–63.5	331–510	0.15–0.59	53.6–115	154–372	0.15–0.36
Lt RenCV (a)	–	–	–	1,834	202	**9.1**
Lt RenCV (b)	–	–	–	1,251	30.2	**41.4**
Lt RenCV (c)	–	–	–	6,357	69.3	**91.7**
RTVs	50.9–178	226–858	0.1–0.23	70–104	470–639	0.13–0.23
Rt. RenCV	–	–	–	60.4	216	0.28

**Figure 2:**
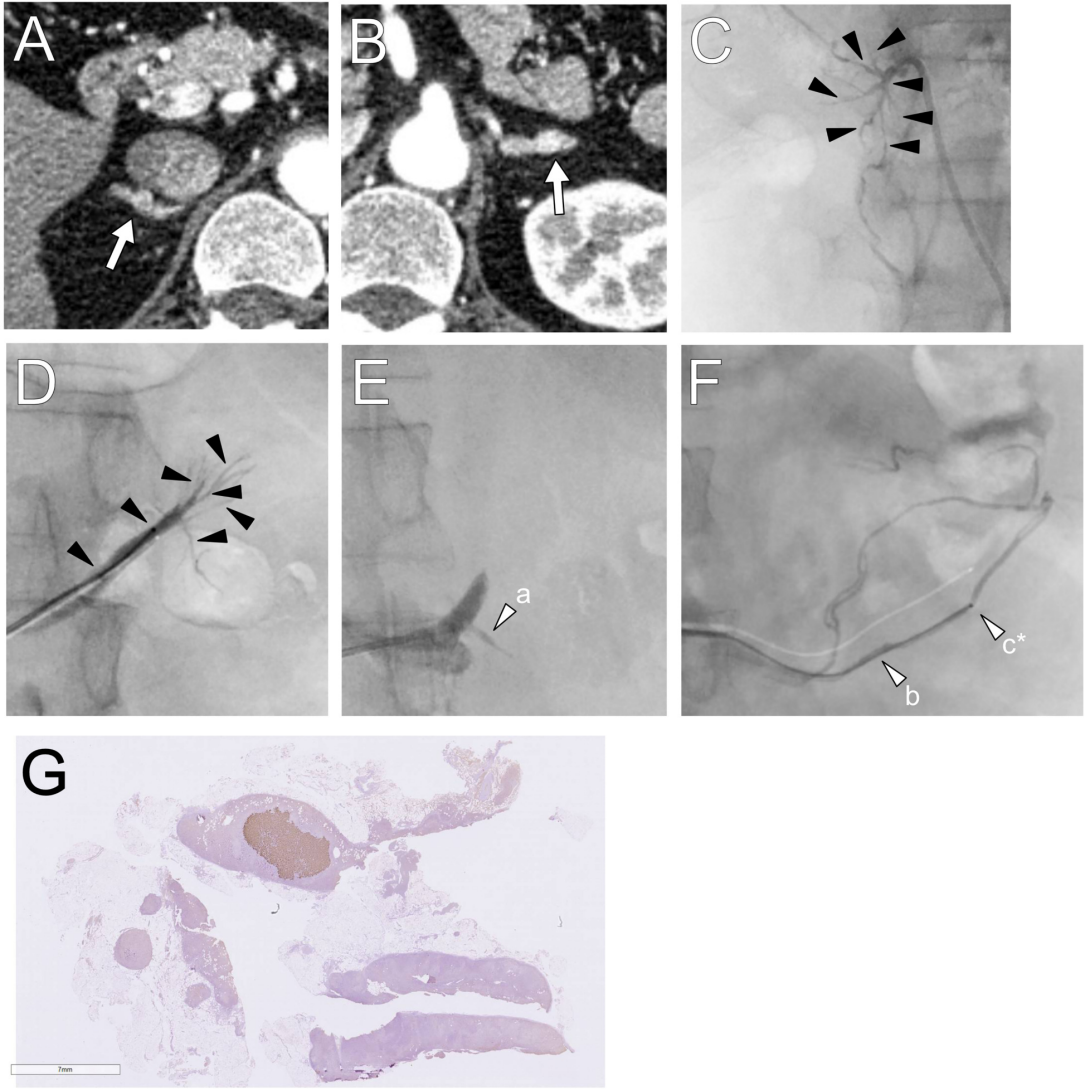
**(A,B)** Computed tomography images showing small adrenal nodules are detected in the bilateral adrenal glands. **(C,D)** In the first session, segmental adrenal tributary venous sampling was performed in the tributaries (black arrowheads). **(E,F)** In the second session, left renal capsular vein sampling was conducted in the fine veins connected to the proximal portion of the adrenal vein and the renal vein (white arrows). The letters a–c in parenthesis shown in Table 1 correspond to sampling points, and those marked with an asterisk indicate the maximum aldosterone concentration. **(G)** Histopathologically, a 6 mm × 4 mm CYP11B2-positive adrenocortical nodule was revealed.

PAC, plasma aldosterone concentration; PCC, plasma cortisol concentration; A/C, aldosterone-to-cortisol; LAV, left adrenal vein; LTV, left tributary; RAV, right adrenal vein; RTV, right tributary; EIV, external iliac vein; LI, lateralization index; RenCV, renal capsular vein. LAV_CV_ and LAV_CMT_ indicate the left adrenal central vein and common trunk, in conjunction with the adrenal central vein and inferior phrenic vein, respectively. LI_CV_ and LI_CMT_ indicate LI using the results of the left adrenal central vein and common trunk on the left side, respectively. The letters a–c in parenthesis correspond to sampling points shown in Figures 1 and 2. The A/C ratio is more than that in EIV indicated in bold.

Initial sAVS was technically successful, and samples were collected from both adrenal veins, several tributaries (Figure 1B), and the left inferior phrenic vein. Table 1 shows the blood sampling results. The selectivity index after ACTH stimulation confirmed successful AVS (2). Aldosterone secretion was mildly predominant on the left side, with a lateralization index [LI; defined as the aldosterone-to-cortisol ratio (A/C) of the dominant side over the contralateral side] of 2.4. The common cutoff for LI is four, and in this case, the result suggested bilateral PA. However, the A/C ratios of the right and left adrenal veins were 0.47 and 1.12, respectively, which were lower than the peripheral A/C ratio of 2.4, indicating apparent bilateral aldosterone suppression (ABAS) or double-down state (5, 8–11). Moreover, the A/C ratio in adrenal tributaries was ≤2.2. This leads to the possibility of anomalous blood drainage from an aldosterone-producing lesion.

Considering abnormal aldosterone-rich blood drainage from the left adrenal cortical adenoma, we reanalyzed the preoperative 0.25-mm-thick CT images using an ultra-high resolution multidetector CT scanner (Aquilion Precision, Canon Medical Systems Corporation, Otawara, Japan), which revealed a barely identifiable fine extra-adrenal vein connected to the adenoma. The total drainage route was not identified, and we attempted to elucidate the route using CT during adrenal arteriovenography (CTAV), which may be used to detect the right adrenal vein during AVS (12, 13) and obtain samples from this vein.

A 5 Fr sheath introducer and a 5 Fr Shepherd hook-shaped diagnostic catheter were inserted from the right femoral artery to identify the left superior and inferior adrenal artery branching from the inferior phrenic artery and accessory renal artery, respectively. Using a 1.7–2.8 Fr tapered microcatheter (Asahi Veloute, Asahi Intecc, Tokyo, Japan) and a 0.016-inch micro guidewire (Asahi Meister, Asahi Intecc, Tokyo, Japan), we cannulated the left superior and inferior adrenal artery and performed CTAV (Figure 1C). The fivefold diluted contrast media was injected at the minimum speed of 0.3 ml/s by a power injector, with scanning starting 10 s after injection began. CTAV images from the inferior adrenal artery showed an enhanced adrenal nodule and a fine renal capsular vein connected to the nodule, draining into the third lumbar vein (Figures 1D,E).

The left third lumbar vein and distal renal capsular vein were cannulated using a 5 Fr Cobra-shaped diagnostic catheter, 2–2.9 Fr tapered split-tip microcatheter (Goldcrest NEO type OM, Medicos Hirata, Osaka, Japan), and micro guidewire from the femoral vein. After ACTH stimulation, blood was sampled from several sites within the vein (Figures 1F,G) under heparinization. Blood sampling required fine positioning due to insufficient blood flow and venous spasms. Finally, blood from the adrenal central vein was collected on the left side. The samples were sequentially submitted to the laboratory for intraoperative measurement, which confirmed elevated aldosterone levels, and the procedure was concluded. The total procedure time was 216 min, with a fluoroscopy time of 67.6 min and radiation dose of 431.2 mGy; the volume of contrast medium used was 77 ml.

The samples showed elevated aldosterone levels in the capsular vein via the lumbar vein, with a maximum PAC of 34,125 ng/dl and an A/C ratio of 146 (Table 1). This led to the diagnosis of left unilateral PA due to a CT-detectable APA, warranting surgery. Additionally, the left adrenal vein PAC and A/C ratio were higher during the second AVS session (2,742 ng/dl and 10.9, respectively) than during the first AVS session. An LI of 23.2 was obtained based on the results of the first right AVS (PAC, 152 ng/dl; A/C ratio, 0.47), which indicated left unilateral PA based on cAVS.

Histopathologically, robotic-assisted left adrenalectomy revealed a CYP11B2-positive aldosterone-producing adrenocortical adenoma (11 mm × 7 mm) (14). Hypertension and hypokalemia were cured, with an ARR of <20, achieving complete biochemical and clinical success 6 months postoperatively, based on the primary aldosteronism surgery outcome (PASO) criteria (15).

### 2.2 Case 2

Preoperative CT revealed small adrenal nodules of ≤6 mm diameter on both sides (Figures 2A,B). sAVS was successfully performed (Figures 2C,D) (2). Table 1 shows the blood sampling results. The LI was 1.1, suggesting bilateral PA; however, the A/C ratios in the adrenal veins and tributaries were ≤0.71; this ratio was lower than that of the iliac vein (1.54). This indicated an ABAS/double-down state (5, 8–11), suggesting anomalous blood drainage of a PA lesion.

A second sAVS was performed, and samples were obtained from the bilateral adrenal tributaries and renal capsular veins connected to the proximal portion of the adrenal vein and renal vein (Figures 2E,F). Intraoperative laboratory testing revealed no elevation of aldosterone levels in the right samples, and the left samples were not examined immediately because it was outside of service hours. This was guided by preoperative contrast-enhanced CT and digital subtraction adrenal venography images, which barely depicted fine renal capsular veins. CTAV was not performed because the highly suspected APA was absent. Blood was also collected from the bilateral adrenal veins. The total procedure time was 284 min, with fluoroscopy time of 67.6 min and radiation dose of 1,144 mGy; the volume of contrast medium used was 85 ml.

The maximum PAC and A/C values in the left renal capsular vein were 6,357 ng/dl and 91.7, respectively (Table 1). The second AVS confirmed left unilateral PA due to an APN, indicating surgery. The PAC in the right or left adrenal central vein and the common trunk (conjunction of the left central vein and phrenic vein) were similar; however, the A/C was relatively higher in the common trunk, with an LI of 5.7.

Robotic-assisted left adrenalectomy was performed, revealing a CYP11B2-positive aldosterone-producing nodule (6 mm × 4 mm) histopathologically (14). Hypertension and hypokalemia were resolved after surgery, with an ARR of <20. The patient achieved biochemical and clinical complete success 6 months postoperatively, based on the PASO criteria (15).

## 3 Discussion

Tumor blood from APAs or APNs might primarily drain out to ADVs rather than the adrenal vein. Herein, we reported two extremely rare cases in which high aldosterone levels were not detected in initial sAVS but were identified in the ADV, specifically the fine renal capsular vein via the lumbar vein and renal vein, including the technical details of their identification. These cases highlight the importance of recognizing anomalous venous drainage routes and the utility of advanced imaging techniques, such as CTAV for precise PA subtyping.

Unilateral APA or APN can be misclassified as bilateral PA through cAVS, because of the following reasons: (1) limited or lack of tumor blood drainage via the adrenal vein; (2) dilution of tumor blood in the adrenal vein due to contamination of normal adrenal blood; (3) technical errors, such as unintentional super-selective sampling beyond the tumor's draining tributary; (4) sampling during the quiescent phase of aldosterone secretion; (5) ectopic APAs; and (6) presence of cortisol-producing lesions, where subtyping with A/C ratio and LI is strongly influenced by cortisol suppression in the adrenal gland (3, 4, 6, 10, 16). sAVS, which includes additional sampling from adrenal tributaries, can diagnose APA and APN more sensitively (3, 4, 6). However, as observed in the current cases, sAVS can still yield false-negative results of APA and APN due to the first reason, although rarely (estimated at <1% of APAs and APNs).

The adrenal gland has an extensive venous network (17–20). Meikos described anatomical extraglandular adrenal veins in addition to the central vein through cadaver studies and revealed that these veins connect to the inferior phrenic vein, adrenal central vein, inferior vena cava, and renal vein (18). These pathways can be visualized via adrenal venography (21) and potentially act as ADV. In fact, previous studies have reported high aldosterone levels in the renal capsular vein, inferior phrenic vein, and lumbar vein in the context of APAs (10, 22, 23). ADV sampling is not always essential for the diagnosis of APAs because some amount of tumor blood usually flows into the adrenal vein. However, as observed in the present study, it may be necessary for diagnosing APA or APN in rare cases.

As the sampling and identification methods for ADVs of APAs remain largely unknown, this report might be highly valuable. Previous reports have demonstrated the utility of CTAV for visualizing the right adrenal vein (12, 13), which is sometimes difficult to cannulate and is a common cause of technical failure during AVS. Case 1 showed the utility of this CTAV for identifying the route of drainage, which was impossible via contrast-enhanced CT. Conversely, in Case 2, CTAV was not utilized because of the presence of bilateral small adrenal nodules on CT; none of these nodules were highly suspected to be APA. This case highlights the possibility of sampling from the anomalous pathways by referring to thin-section CT images and adrenal venography. However, detection of small vessels at approximately 1–2 mm in size on imaging, as well as cannulation and sampling, is challenging.

Predicting the need for ADV sampling before or during AVS is difficult. Even when an ADV appears connected to the tumor on CT, adrenal vein or tributary sampling often captures nearly all APA-derived blood. Rapid aldosterone and cortisol measurements can help verify whether tumor blood is present in the sample and assist in determining the intraprocedural endpoint during a second AVS, as demonstrated in the present cases, and potentially during the first AVS, although this approach requires additional time. In the ABAS or double-down state, where the A/C ratio is lower in the bilateral adrenal venous blood than in peripheral blood, ADV sampling should be considered to be performed after the first AVS because one of the causes is tumor blood drainage into the ADV. DePietro et al. (5) described 10 patients with APAs who exhibited ABAS in the first cAVS and underwent repeated AVS. Among these patients, three showed bilateral PA twice. Thus, further examination, such as sampling from ADVs or sAVS, might be needed for accurate subtyping. To our knowledge, ADV sampling is technically challenging and rarely necessary; therefore, sAVS, which is easier to perform, is preferable.

However, the seven remaining patients had unilateral PA with an LI of ≥3.5 from repeated cAVS, with caution of incidental deep sampling (5). Thus, only repeated precise cAVS alone might be sufficient for accurate subtyping. We routinely perform segmental AVS and ensure not to go beyond the branching points of all tributaries, making technical causes unlikely. Nevertheless, the calculated LI based on the second AVS becomes higher than on the first AVS. However, it has no supporting evidence. In Case 1, the LI of 23.2 in the second AVS indicated unilateral PA. However, the vasospasm of the renal capsular vein during sampling might have altered the drainage pathway of the APA blood to the adrenal vein, which blood was sampled after the onset of the vasospasm. In Case 2, the LI was 1.8 and 5.7 based on the results of the left adrenal central vein and the common trunk, the conjunction between the left adrenal central vein and inferior phrenic vein, respectively. The LI of 5.7 indicated unilateral PA; however, equal levels of aldosterone in the left common trunk and right adrenal veins (PAC, 117 and 139 ng/dl, respectively) might cause hesitation in determining unilateral PA and indication for surgery. Thus, the detection of high aldosterone levels in ADV would be particularly beneficial to determining the treatment plan.

In ABAS cases, other possible causes, which are similar to the reasons for APA and APN misclassification, should also be considered, except for the cortisol-producing lesion mentioned above. Cases of blood sampling during a quiescent period of aldosterone secretion reportedly have decreased with ACTH stimulation (9–11). Therefore, repeated cAVS under ACTH stimulation should be considered. The possibility is unintended super-selected sampling, which is a technical issue where blood is sampled beyond the branches where tumor blood is being discharged. Anatomical variations, such as duplicate adrenal veins, should also be considered (17, 24, 25). The possibility of ectopic APAs could exist but it is extremely rare.

In recent years, non-invasive lateralization techniques such as ^11^C-metomidate positron emission tomography (PET) and ^68^Ga-Pentixafor PET/CT have been investigated at select centers (26, 27). For APAs ≥1 cm, ^68^Ga-Pentixafor PET has demonstrated high sensitivity, with a detection rate of 97.3% (36/37) (27). However, false negatives for APAs and false positives for non-functioning adrenal adenomas can still occur. A concordance rate of 77% between ^68^Ga-Pentixafor PET and AVS findings has been reported (28), indicating that neither modality is definitively superior. Similar to other PET tracers, PET/CT imaging is limited by spatial resolution. Although PA lesions smaller than 1 cm can occasionally be detected, the detection rate for subcentimeter lesions was only 60% (3/5) (27), indicating potentially lower sensitivity for small lesions. Access to these advanced imaging techniques remains extremely limited, including at our institution. Although the approach presented in this report is not necessarily simple, it is considered feasible and clinically valuable.

In conclusion, sampling from ADVs is rarely essential for the accurate diagnosis of APAs and APNs. Moreover, CT during adrenal arteriovenography might be useful for precisely estimating the drainage routes.

## Data Availability

The raw data supporting the conclusions of this article will be made available by the authors, without undue reservation.
